# The experience and reflections of GC labs as an independent clinical laboratory to the COVID-19 pandemic in South Korea

**DOI:** 10.1186/s12879-023-08684-0

**Published:** 2023-12-06

**Authors:** You La Jeon, Sang Gon Lee, Eun Hee Lee, Sungwook Song, O-Jin Lee, Un Yeong Go, Ga-Young Chun, Hyun Mi Choi, Jin Young Choi

**Affiliations:** Department of Laboratory Medicine, Green Cross Laboratories, 107, Ihyeon-Ro 30Beon-Gil, Giheung-Gu, Yongin-Si, Gyeonggi-Go South Korea

**Keywords:** COVID-19, SARS-CoV-2, Control, Laboratories

## Abstract

**Background:**

Amid the COVID-19 pandemic, extensive testing was undertaken by independent clinical laboratories (ICLs), yet limited research exists on this matter. Drawing from Green Cross Laboratories (GC Labs)' pandemic response experience, this study seeks to offer insights for preparation for the next pandemic.

**Methods:**

This retrospective study analyzed the outcomes of SARS-CoV-2 real-time reverse transcription polymerase chain reaction (SARS-CoV-2 rRT PCR) tests administered by GC Labs for COVID-19 diagnosis, upon request by different organizations, between February 2020 and April 2022. The distribution of institutions that requested the tests, the type of tests, and the positive rate were analyzed. We investigated resource allocation details.

**Results:**

ICLs were responsible for conducting 85.6% of all tests carried out under South Korea’s COVID-19 testing policy during the pandemic. The availability of free testing regardless of symptoms led to a significant increase in the use of pooled tests, which accounted for more than 80% of all tests conducted after August 2021. The gender and age distribution of COVID-19 cases nationwide and GC Labs’ positive cases were similar. When we analyzed the positive rate by requesting organizations during the COVID-19 pandemic, despite an overall nationwide positivity rate of 35%, high-risk facilities exhibited a positivity rate of less than 5% by maintaining preemptive testing. The most notable increase in resources during the pandemic was seen in human resource input.

**Conclusions:**

South Korea's ICLs were able to conduct large volumes of testing during the COVID-19 pandemic because of their logistics and computer systems, scalable testing space, and trained testing personnel. They also had the flexibility to bring in additional resources to expand testing capacity because they are specialized testing organizations. Hence, ICLs could execute the pooled test that the government had introduced for extensive general population screening. The preemptive periodic testing of high-risk populations kept the positive rate much lower than in the general population. This study's findings will aid in refining mass testing-based policies for the next pandemic.

## Background

Since the World Health Organization declared COVID-19 a pandemic on March 11, 2020, more than 750 million cases of COVID-19 have been reported globally, resulting in over 6 million deaths as of February 16, 2023 [1, 2]. In South Korea, the number of identified cases has exceeded 30 million, with 34,000 deaths, as of February 16, 2023 [3].

At the outset of the pandemic, testing was the primary focus due to the absence of appropriate treatment or a vaccine. The WHO emphasized that testing, isolation, and contact tracing were critical elements of the global pandemic response [4]. In South Korea, the initial outbreak was successfully contained through the 3 T strategy (testing, tracing, and treatment). Testing is a crucial tool for identifying suspected COVID-19 cases, confirming infection in contacts, and determining the release of individuals from quarantine [5–9].

Since the beginning of the pandemic, nucleic acid amplification tests such as SARS-CoV-2 real-time reverse transcription polymerase chain reaction (SARS-CoV-2 rRT PCR) have been used as the reference standard for COVID-19 diagnosis [10]. In South Korea, 77.56 million SARS-CoV-2 rRT PCR tests were performed before the rapid antigen test (RAT) by clinicians was approved for confirmation on March 14, 2022. As of June 15, 2022, the number of SARS-CoV-2 rRT PCR tests exceeded 100 million [11].

South Korea’s Public–Private Partnership facilitated the rapid introduction of reagents approved through the emergency use authorization (EUA) system in clinical laboratories [8, 12, 13]. It enabled reagent manufacturers to meet the rising demand for testing. Furthermore, to address the worsening COVID-19 epidemic, testing was expanded to a broader range of subjects, pooled specimen tests were introduced to increase capacity, and screening policies for entrants from abroad were strengthened.

During the COVID-19 pandemic, independent clinical laboratories (ICLs) performed 86% of all SARS-CoV-2 rRT PCR tests in South Korea. The big ICLs in South Korea had their own nationwide logistics networks and systems in place to report test results electronically. Furthermore, when juxtaposed with university hospitals, general hospitals, or public clinical laboratories, these ICLs already possessed more expansive testing environments and trained testing personnel. This advantageous setup enabled them to promptly adapt to government testing policies and conduct robust testing under government contracts. Meanwhile, hospitals had to reallocate additional resources, such as physical space and medical staff, to accommodate both COVID-19 patients and those with other medical conditions [12].

This study aimed to investigate the role and response of ICLs during a large-scale testing initiative for COVID-19 in South Korea, based on the experience of Green Cross Laboratories (GC Labs). Additionally, during the COVID-19 pandemic, an analysis of relevant data such as the types of referral institutions and tests that requested SARS-CoV-2 rRT PCR tests from GC Labs, as well as the characteristics of positive cases, was conducted to assess how the testing policies were reflected in such data.

## Methods

### Study period

This study retrospectively analyzed the results of SARS-CoV-2 rRT PCR tests requested by various organizations to GC Labs to diagnose COVID-19. In this study, individual tests were conducted from February 7, 2020, to April 30, 2022, and pooled tests were conducted from May 26, 2020, to April 30, 2022. The results of SARS-CoV-2 rRT PCR tests for research purposes were excluded.

### Study design

First, we analyzed how the proportion of each type of SARS-CoV-2 rRT PCR test and requesting institution changed due to the governmental testing policies. The test was divided mainly into two types: individual tests and pooled tests. The requesting organizations were classified as follows: screening stations of public health centers, temporary screening stations of public health centers, medical institutions, high-risk facilities, temporary residential facilities, and residential treatment centers.

Screening stations of public health centers requested individual tests for those who had symptoms, contact with confirmed cases, or epidemiological relevance to domestic outbreaks. In temporary screening stations of public health centers, starting with anonymous testing in December 2020, free testing was allowed regardless of symptoms, epidemiological relevance, or region, and most requests were for pooled tests. However, as the number of test subjects exploded in February 2022, the functions of screening stations and temporary screening stations were identical. To manage high-risk facilities vulnerable to infection, such as nursing hospitals and nursing homes, periodic testing was implemented for all workers and patients or residents beginning in October 2020. Temporary residential facilities allowed entrants from abroad who did not have a residence in South Korea to receive a COVID-19 test and be isolated for a certain period after entering the country. Residential treatment centers were places where patients with mild symptoms and a low need for hospitalization could receive isolation treatment for a specific time, and these centers were actively operated in the early stages of COVID-19.

Information on testing policies and vaccination was obtained from the COVID-19 response guidelines (~ 13th edition) and procedures for the COVID-19 vaccination certificate and COVID-19 negative confirmation system (~ 3rd edition) published by the South Korea Diseases Control and Prevention Agency (KDCA). Data on the number of COVID-19 PCR tests performed in South Korea were obtained through a request through the information disclosure system to the KDCA.

Second, we analyzed the trend in the incidence of confirmed COVID-19 cases nationwide and at GC Labs and compared the epidemiological characteristics of COVID-19 cases. For the epidemiologic features of COVID-19 cases nationwide, we used KDCA’s COVID-19 domestic infectious disease occurrence data.

Third, we analyzed the response of how GC Labs, a big ICL in South Korea, expanded its capabilities to enable robust testing in coping with the COVID-19 pandemic. We reviewed GC Labs’ internal approval documents to investigate resource allocation details, such as equipment, human resources, and testing space.

### Statistics

Data were analyzed using SAS (version 9.4, SAS Institute, Inc., Cary, NC, USA). COVID-19 case numbers are expressed as discrete values, while categorical variables such as gender, age, and requesting institution were reported as percentages. Pearson chi-square test was used for the comparison between epidemiological characteristics of COVID-19 cases nationwide and at GC Labs. The number of tests performed by GC Labs and the number of additional resources assigned were expressed in multiples based on the number of tests performed in March 2020.

## Results

After RAT tests by clinicians were recognized as confirmatory tests starting on March 14, 2022, the demand for PCR testing significantly decreased. Consequently, the study period for this analysis was set from February 2020 to April 30, 2022, as there was a notable shift in testing requests during this period.

### Analysis of SARS-CoV-2 rRT PCR test request status

**Fig. 1:**
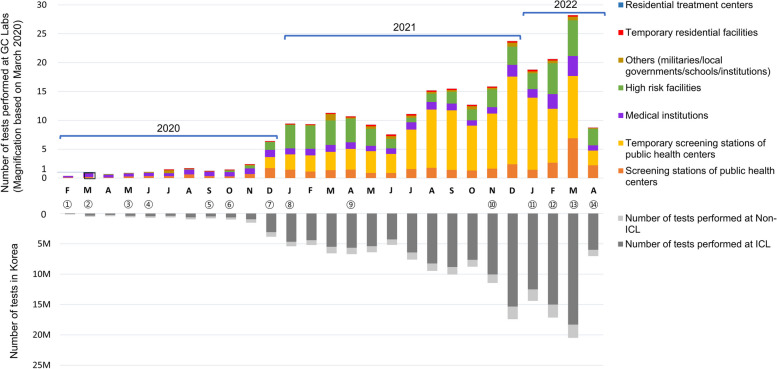
Analysis of the request status of the SARS-CoV-2 rRT PCR test. The number of tests performed at GC Labs represented magnification based on March 2020. ① Development of SARS-CoV-2 molecular diagnostic test by KDCA (January 31). MFDS approved the first EUA of SARS-CoV-2 rRT PCR (February 4). Beginning of SARS-CoV-2 rRT PCR use in general medical institutions, including independent clinical laboratories (ICLs) (February 7). ② Criteria for release from isolation according to the presence or absence of symptoms of a confirmed case (March 2).—Symptomatic patients: Satisfaction with both clinical course-based criteria and test-based criteria (negative result twice consecutively at 24-h intervals) without lapsed time. ③ Protocol for SARS-CoV-2 rRT PCR pooling test (May 1). Mandatory SARS-CoV-2 rRT PCR for all arrivals (May 11). Operation of COVID-19 screening station (May 15). Reinforcement of infection prevention and management for high-risk facilities and vulnerable infectious facilities.—preemptive tests (May 15). Extension of isolation period for confirmed cases 7 days (May 11) ➔ 10 days (June 25). ④ Criteria for release from the isolation of symptomatic patients – even if they meet only clinical course-based criteria (June 25). ⑤ Start of prehospitalization pooled test (September 21). ⑥ Tests for all workers and caregivers in vulnerable infectious facilities and patients or residents of adult day care centers throughout the nation (October 19). ⑦ Expansion of preemptive diagnostic tests (December 10).—Reinforcement of testing for workers and caregivers in nursing hospitals and nursing homes nationwide. Opening free tests at temporary screening stations regardless of the presence of symptoms or epidemiologic association for all citizens (when social distancing level 2 or higher). ⑧ Active recommendation of SARS-CoV-2 rRT PCR to subjects with positive rapid PCR or rapid antigen test (RAT) results (January 22). SARS-CoV-2 rRT PCR within one day and additional PCR test on the 13.^th^ day after entry for all arrivals (January 22). ⑨ Free tests available to all citizens without restrictions on social distancing steps (April 12). ⑩ Introduction of proof of vaccination or PCR negative confirmation system (Quarantine pass) following the strategy for step-by-step daily recovery transition (November 1). ⑪ Apply RAT as quarantine pass (January 26). ⑫ Reorganization of the COVID-19 diagnostic test system in preparation for Omicron epidemic (February 3) – Priority designation of PCR test application, RAT for other applicants. Reduction of isolation period for confirmed cases (February 10). ⑬ Temporary suspension of quarantine pass (March 1). Recognition of RAT performed by a clinician as a confirmatory test of COVID-19 (March 14). ⑭ Suspension of free RAT at the screening station of public health centers (April 11). Adjusted COVID-19 from class 1 infectious diseases to class 2 infectious diseases (April 25)

Based on South Korea’s COVID-19 test results, 85.6% of all tests conducted in the country were performed by ICLs. Initially, the proportion of tests conducted by ICLs was less than 70%. Since December 2020, free testing has been offered to all citizens as a preemptive diagnostic measure, resulting in a substantial increase in the fraction of tests by ICLs to nearly 90%, as illustrated in Fig. 1. Non-ICLs include clinical laboratories in general and university hospitals and laboratories belonging to KDCA.

Initially, during the early stages of the COVID-19 outbreak, a significant proportion of the test requests were made by medical institutions. However, as the pandemic progressed, public health centers increased their sampling capacity and started monitoring high-risk facilities from October 2020 onward. Consequently, by December 2020, approximately 80% of the test requests made to GC Labs were from screening stations, temporary screening stations, and high-risk facilities.

**Fig. 2:**
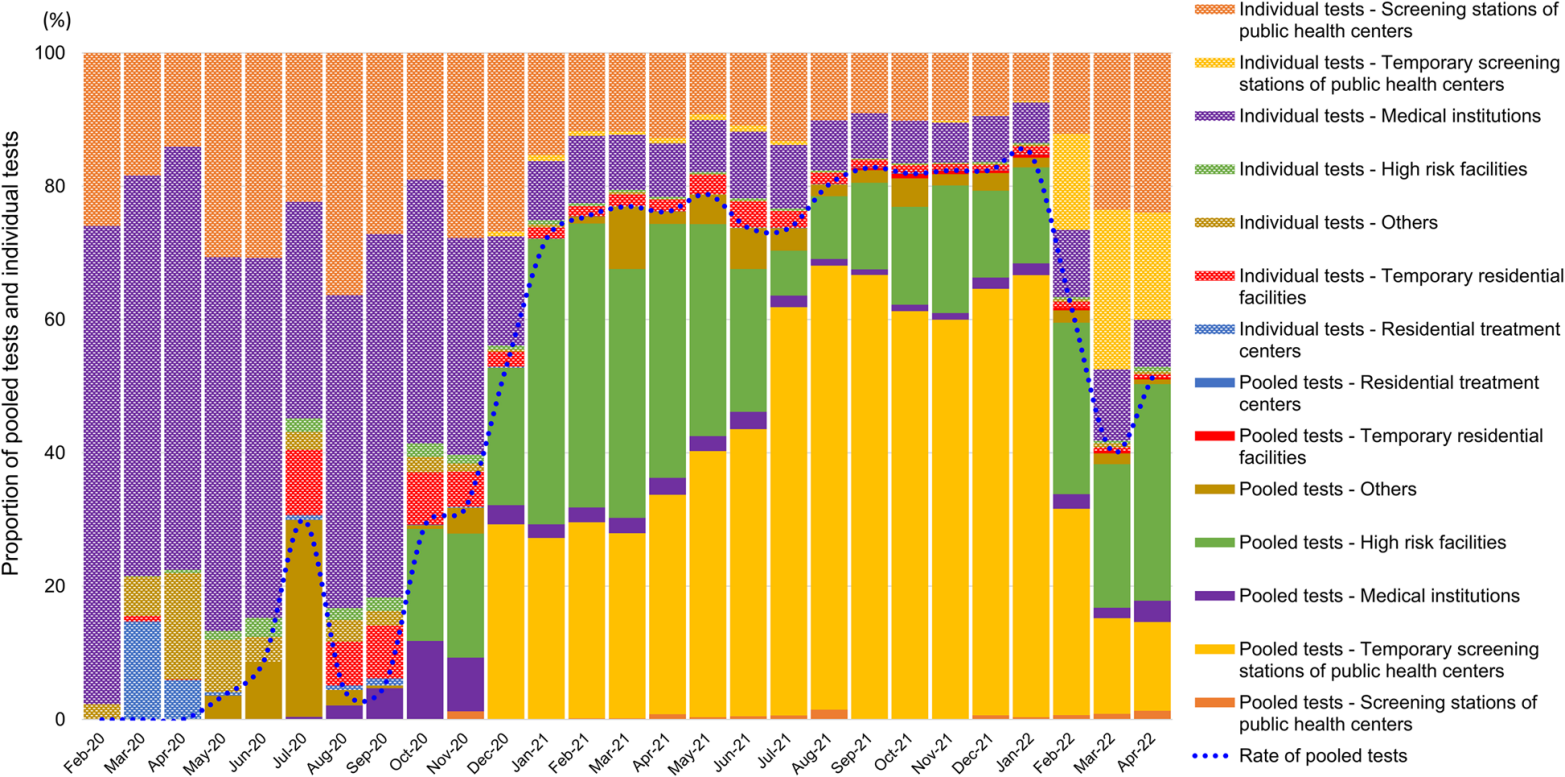
The proportion of individual and pooled tests requested by the requesting institutions. In October 2020, monitoring for workers and users of high-risk facilities using periodic pooled tests was started. With the free testing that began in December 2020, the percentage of captured tests exceeded 50%; by August 2021, it was over 80%

Figure 1 depicts the testing status of nationwide ICLs and the request status of GC Labs by requesting institutions from February 2020 to April 2022, with significant policy changes described in the caption. Figure 2 shows the proportion of pooled and individual tests requested by requesting institution type. During the early stages of the pandemic, medical institutions primarily ordered individual tests with some pooled tests to confirm COVID-19 before hospitalization. However, as of October 2020, public health centers expanded their sampling capacity and began monitoring high-risk facilities. As a result, high-risk facilities began conducting periodic pooled tests for workers and users. In December 2020, free testing was made available to the public regardless of symptoms, leading to pooled test requests exceeding 50% and subsequently exceeding 80% in August 2021. The increase in testing of contacts due to the rise in confirmed cases since February 2022 led to a greater proportion of individual tests being conducted.

### Positive cases of SARS-CoV-2 rRT PCR

**Fig. 3:**
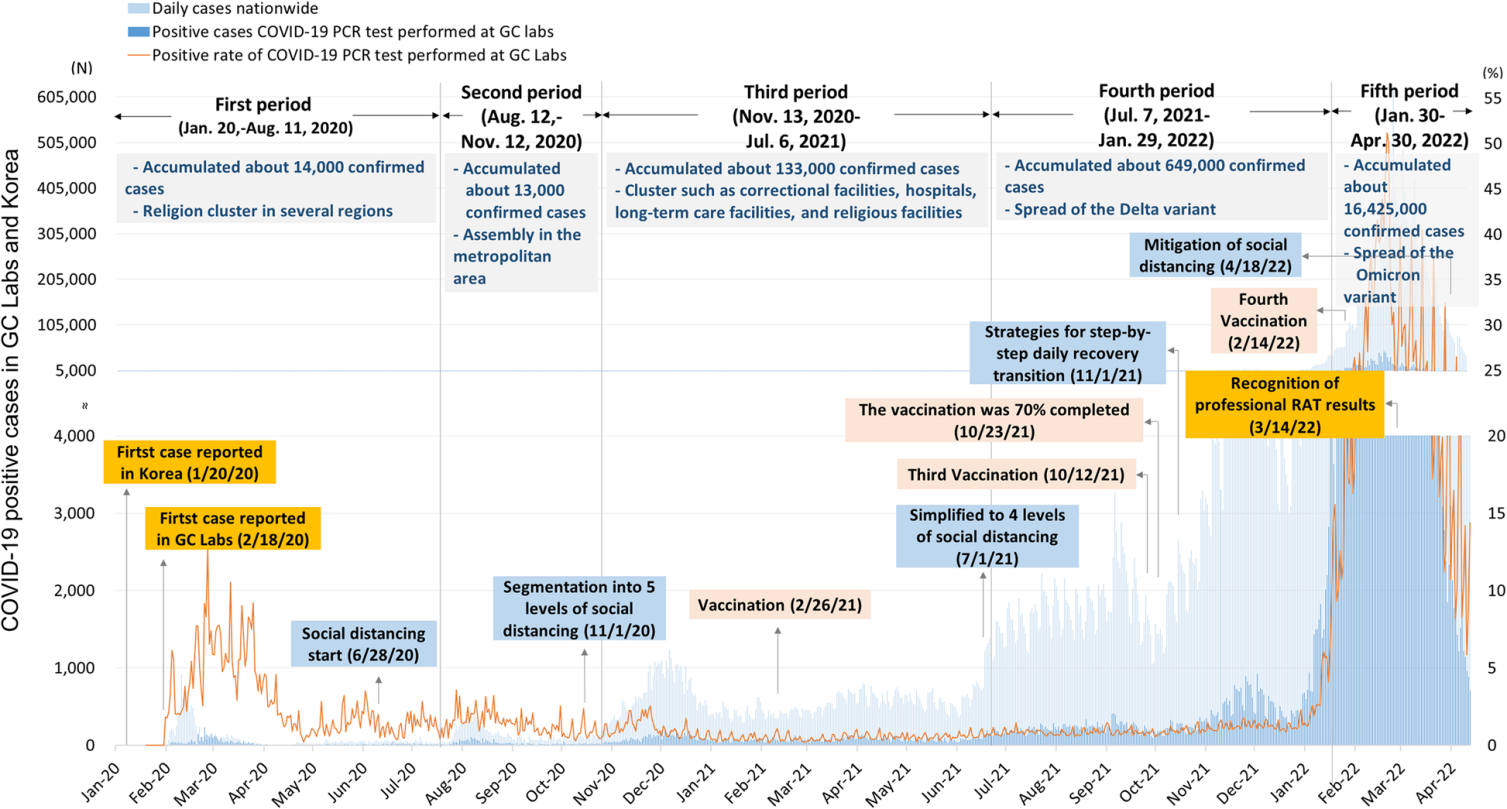
The COVID-19 cases nationwide and at GC Labs

Figure 3 shows the number of COVID-19 cases nationwide in South Korea and GC Labs across five distinct periods of COVID-19 progress.

**Fig. 4:**
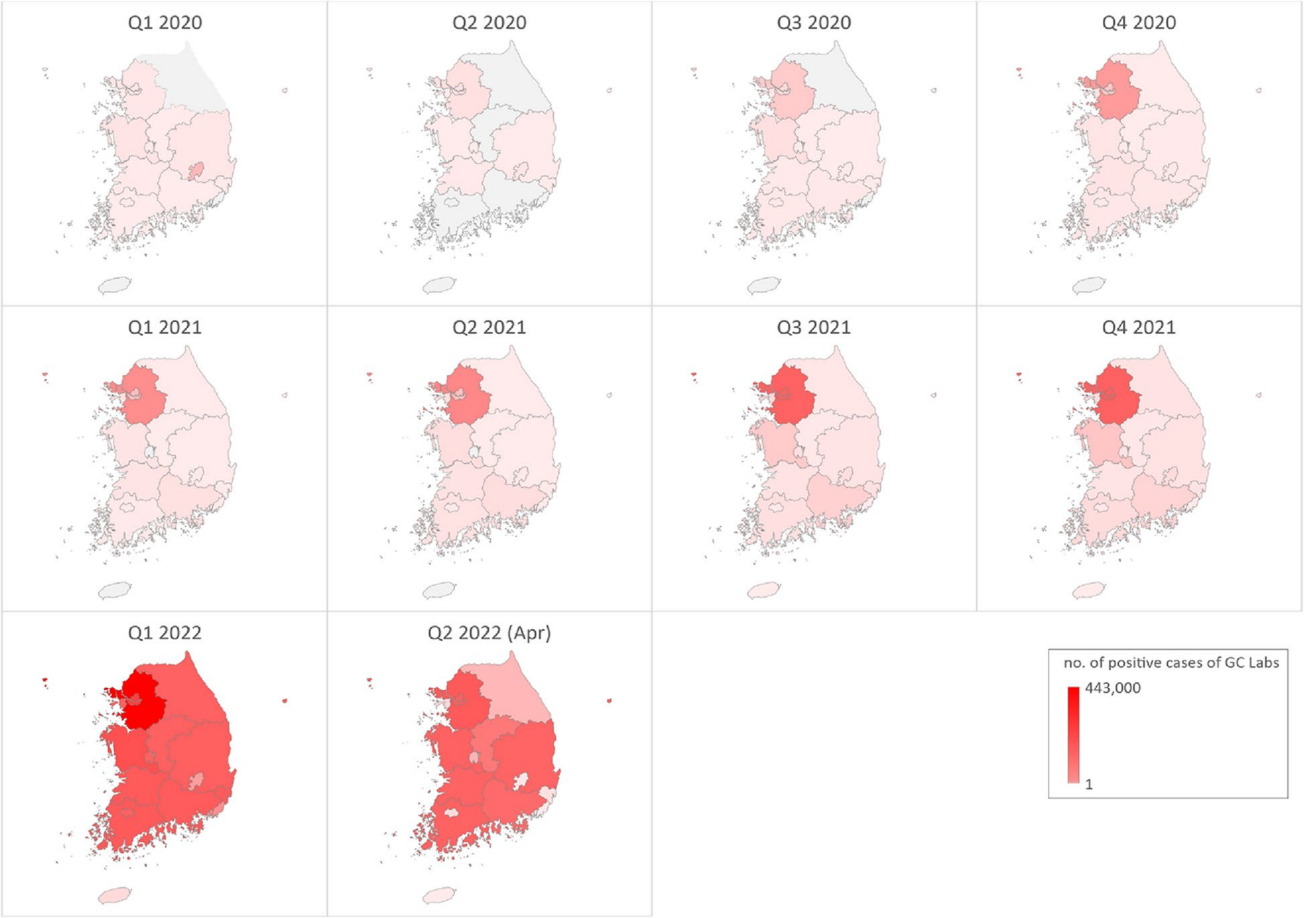
The COVID-19 nationwide outbreak map of Korea based on GC Labs data. In the first quarter of 2020, the outbreak centered on religious clusters in the Daegu and Gyeongbuk regions. Since then, many positive cases have occurred in large, densely populated cities

The first period, which ended on August 11, 2020, was characterized by a large-scale epidemic stemming from religious clusters in Daegu and Gyeongbuk (Fig. 4). During this time, the positive rate temporarily surged to 13%. The second period, spanning from August 12, 2020, to November 12, 2020, saw the occurrence of some assemblies of various sizes. On November 1, 2020, the South Korean government implemented a five-stage social distancing system.

The third period covered the timeframe from November 13, 2020, to June 6, 2021, and was marked by sporadic outbreaks in religious, nursing home, and prison settings. In February 2021, vaccination efforts were initiated for users and workers under the age of 65 in high-risk facilities such as nursing hospitals and homes. During the fourth period, the epidemic expanded further due to the Delta variant, resulting in over 5,000 confirmed cases occurring daily, and predominantly affecting metropolitan areas, including Gyeonggi and Seoul, until the fourth quarter of 2021. However, the Omicron mutations led to the nationwide spread of infections, as shown in Fig. 4. In the fifth period, the spread of the Omicron mutation resulted in up to 600,000 confirmed cases occurring daily, with GC Labs reporting a positive rate exceeding 50%. Since March 14, 2022, professional RAT results have been accepted, leading to a significant decrease in the number of SARS-CoV-2 rRT PCR tests conducted and the number of positive reports.

During the study period, when comparing the incidence rates of COVID-19 cases nationwide and those at GC Labs based on gender and age groups, statistically significant differences were observed (*P* < 0.0001) (Table 1). However, when dividing the study period into five intervals, a consistent trend emerged where the proportion of positive cases increased from the first to the fifth interval. Additionally, in both nationwide and GC Labs' COVID-19 cases, the proportion of females exceeded that of males. Furthermore, the distribution pattern based on age groups exhibited a similar trend in both datasets. The highest proportion was observed in the 40 s age group, followed by the 30 s, 20 s, 10 s, 50 s, under 10 s, 60 s, 70 s, and 80 s and above groups, with a gradual decrease in proportions.

**Table 1:** Comparison of the COVID-19 cases nationwide and at GC Labs

Characteristics	GC Labs	Nationwide	*P*- value
	Number	Number	
	(%)	(%)	
Total number	1,136,159	17,234,660	
(January 2020 – April 2022)	(100%)	(100%)	
Distribution of COVID-19 cases			< 0.0001
First period			
(Jan. 20, -Aug. 11, 2020)	4,399 (0.39%)	14,660 (0.09%)	
Second period			
(Aug. 12, -Nov. 12, 2020)	2,913 (0.25%)	13,280 (0.08%)	
Third period			
(Nov. 13, 2020 – Jul. 6, 2021)	19,541 (1.72%)	133,591 (0.78%)	
Fourth period			
(Jul. 7, 2021 – Jan. 29, 2022)	73,860 (6.50%)	649,238 (3.77%)	
Fifth period			
(Jan. 30,—Apr. 30, 2022)	1,035,446 (91.14%)	16,423,891 (95.30%)	
Sex rate (male: female)	1: 1.05	1: 1.13	< 0.0001
Composition by age group			
0–9	132,985 (11.70%)	2,100,118 (12.19%)	< 0.0001
10–19	146,174 (12.87%)	2,263,022 (13.13%)	
20–29	154,441 (13.59%)	2,476,698 (14.37%)	
30–39	165,012 (14.52%)	2,538,524 (14.73%)	
40–49	175,284 (15.43%)	2,668,201 (15.48%)	
50–59	135,407 (11.92%)	2,119,428 (12.30%)	
60–69	115,887 (10.20%)	1,745,665 (10.13%)	
70–79	52,745 (4.64%)	822,371 (4.77%)	
≥ 80	38,766 (3.41%)	500,633 (2.90%)	
unknown	19,458 (1.71%)	-	

**Fig. 5:**
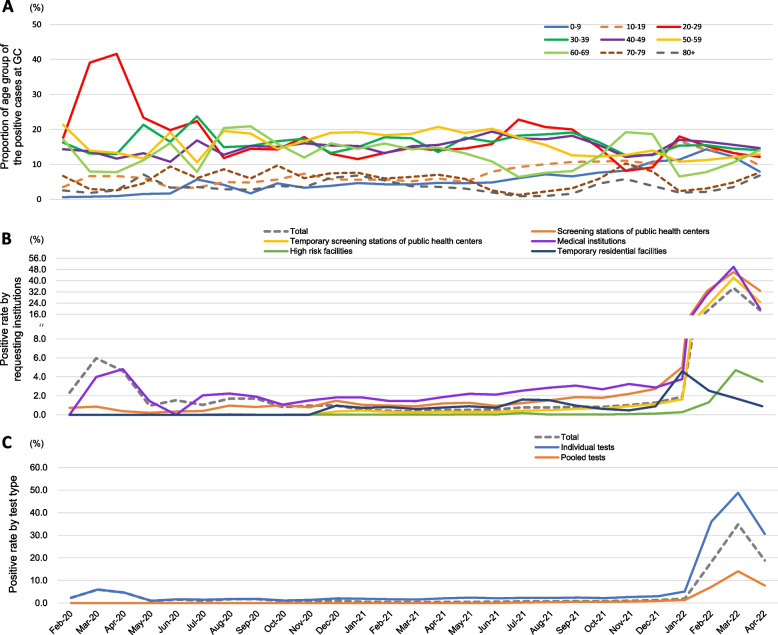
The incidence trends of COVID-19 cases during the study period at GC Labs. **A** shows the proportion by age group among GC Labs’ positive cases during the COVID-19 pandemic. The positive rate for high-risk facilities peaked around the same time as the positive rate for other institutions but was within 5% (**B**). The positive rate for temporary residential facilities for arrivals peaked in January 2022, reflecting the trend of other countries. **C** shows the positive rate according to individual or pooled tests

Figure 5 presents a comprehensive analysis of the incidence trends of COVID-19 cases during the study period at GC Labs. It should be noted that only GC Labs’ positive results were available for analysis regarding the proportion by age among GC Labs’ positive cases during the COVID-19 epidemic (Fig. 5A). In March–April 2020, the proportion of people in their 20 s was higher than 40% due to the influence of the religious cluster. From the study period, the age range of 20–69 years accounted for 10%-20% of COVID-19 cases. Children aged 0–9 and 10–19 represented less than 10% of COVID-19 cases until May 2021, but their proportion gradually increased. Conversely, those over 70 consistently accounted for a low proportion during the study period. Regarding the positive rate by requesting institutions, medical institutions had a notably high positive rate in March–April 2020 (Fig. 5B). However, from December 2021, the positive rate of screening stations and temporary screening stations of public health centers and medical institutions rapidly increased, reaching 50% in March 2022. High-risk facilities also had peak positive rates around the same time but remained under 5%. Temporary residential facilities reflected the situation in other countries and had the highest positive rate in January 2022, earlier than South Korea’s peak in March 2022. Analyzing the trend of positive rates by individual and pooled tests revealed that individual tests reflected the rapid increase in positive rates (Fig. 5C).

### Resource input analysis

**Fig. 6:**
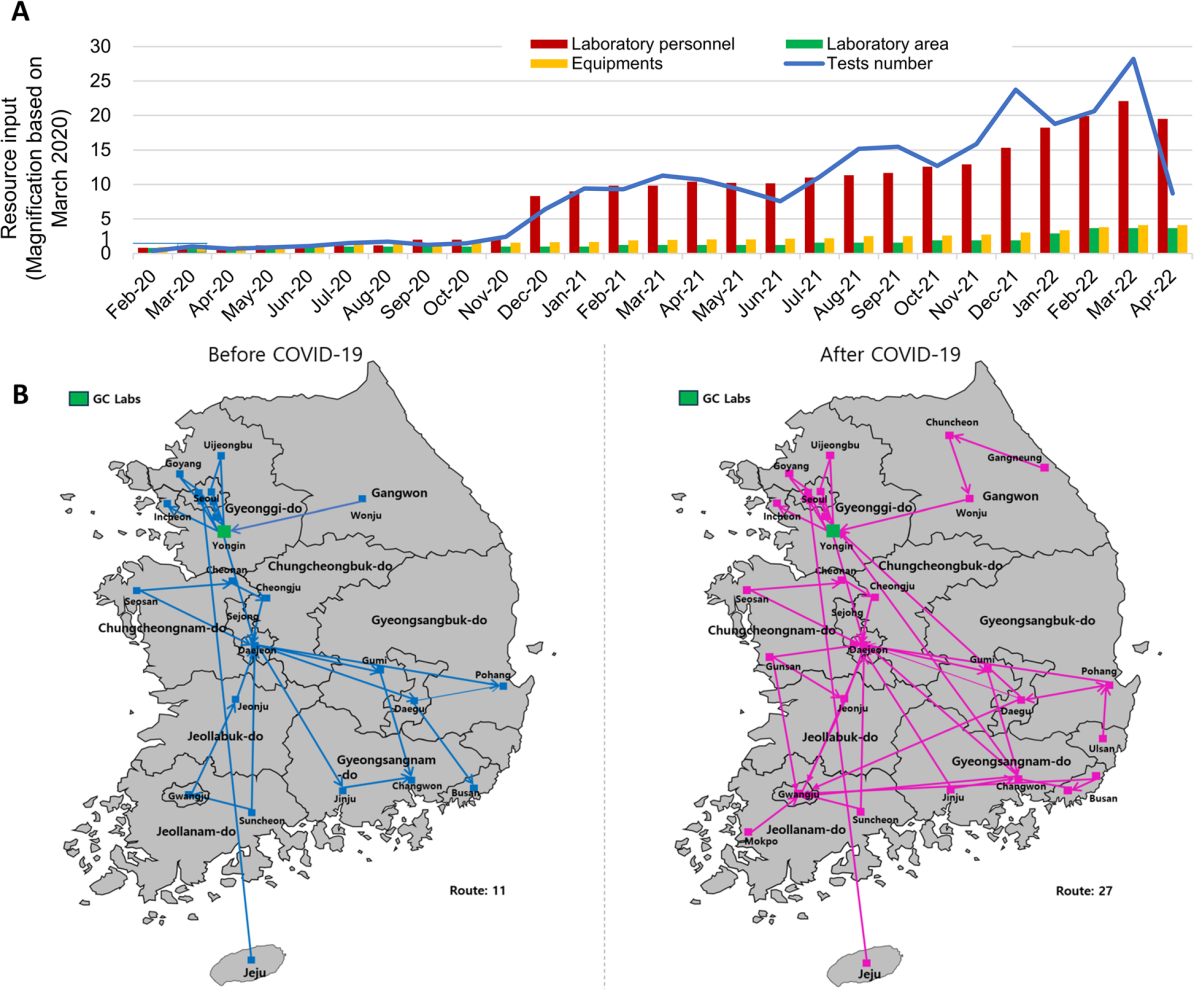
Resources input in response to the COVID-19 pandemic in GC Labs. The amount of resources is shown as magnification based on March 2020 in **A**. Human resource input was mainly affected by the number of tests among various resources. **B** shows the expansion of the logistics network for sample transportation at GC Labs

The resource input analysis in Fig. 6 depicts the escalation of testing space, equipment, and human resources for conducting SARS-CoV-2 rRT PCR tests correlated with changes in the number of tests performed. The sensitivity of human resource input was particularly pronounced relative to the number of tests. In January 2021, test numbers rose by nearly tenfold compared to March 2020, and our laboratory allocated a corresponding tenfold increase in human resource input. Adequate human resources were assigned in response to the significant increases in testing volume in August and December 2021, and March 2022. With the more than 28-fold increase in test numbers by March 2022, the human resources input increased by 22-fold, and the number of tests processed per medical technician increased by over 1.2-fold. In contrast, testing space or equipment increases were more gradual, with an input increase of up to fourfold. Since the onset of the COVID-19 outbreak, our logistics network has undergone expansion, encompassing a total of 27 routes, a substantial increase from the initial 11 routes.

## Discussion

In response to the pandemic, a variety of public–private partnerships have been established, encompassing a range of initiatives, including vaccine development, personal protective equipment provision, allocation of medical resources, surveillance program implementation, and expansion of laboratory capacity to manage the sharp rise in testing demand [14]. Among these endeavors, the present study specifically focuses on the contribution of ICLs to the implementation of the government’s pandemic response policy.

During the early stages of the COVID-19 epidemic, South Korea conducted extensive testing by rapidly introducing high-performance reagents into clinical laboratories through the EUA system and expanding testing capacity at governmental and nongovernmental levels [13]. The government adjusted its testing policy based on the epidemic, changing the requesting institutions or request types. This study examined the status of SARS-CoV-2 rRT-PCR tests performed over two years and three months, beginning with the first COVID-19 case in South Korea at GC Labs, one of the largest ICLs. These findings are crucial for developing a testing policy for emerging infectious diseases, with this study being the first to investigate the matter.

The time frame spanning from the first COVID-19 case in January 2020 to April 2022 can be delineated into five periods. Notably, the point at which test volume surged was in December 2020, when free testing became available to all citizens. Before this, there was little discrepancy in the contribution of ICLs and non-ICLs (e.g., general medical institutions or governmental laboratories) to screening, with ICLs accounting for over 60% of tests [15]. Subsequently, ICLs emerged as distinct contributors to South Korea’s robust testing capacity. In December 2020, as free testing was made available to the general public irrespective of symptoms or contact with COVID-19 cases, test volume increased nearly threefold compared to the previous month, with ICLs performing almost 80% of all tests conducted (Fig. 1).

In May 2020, the Korean Society of Laboratory Medicine (KSLM) issued a protocol for pooled testing [16]. As a result, pooled tests have gradually increased, particularly for prehospital testing in medical institutions and routine screening of high-risk facility workers and patients or residents. Since December 2020, pooled tests have accounted for 50–80% of all tests conducted (Fig. 2). It should be noted that mixing samples in pooled tests can only be accomplished manually, making it a time-consuming task that cannot be significantly expedited even with improved technical expertise. Consequently, proper allocation of human resources has become the most critical factor since then.

With the emergence of the Delta mutation as the dominant strain in July 2021, the test volume again experienced a notable increase [17]. Following a step-by-step daily recovery policy incorporating a quarantined pass, the number of tests peaked in March 2022 following the emergence of the Omicron mutation [18]. During this period, the number of confirmed COVID-19 cases in South Korea was at its highest, resulting in the largest number of requests for screening at public health center stations focused on contact tracing (Fig. 1).

The positive rates by gender and age group in South Korea and GC Labs showed similar trends. Looking at the change in the positive rate by age group during the pandemic with data from GC Labs, those aged 20–69 who are active outside comprised a relatively high proportion of COVID-19 cases. When the decline in academic performance due to the continuation of non-face-to-face classes due to COVID-19 was visibly confirmed, face-to-face classes were expanded from June 2021. As a result, the proportion of school-age children in COVID-19 cases showed a slight increase.

When analyzed by type of requesting institution, the positive rate of high-risk facilities was within 5% even in March 2022, when the total positive rate reached 35% due to the influence of Omicron mutation. This shows that the management of high-risk facilities was thorough [19]. Conducting preemptive testing in high-risk facilities, regardless of the occurrence of infection, can lower the overall prevalence [20]. Given that the prevalence of COVID-19 in long-term care facilities in other countries is reported to be higher than that of the general population, it can be seen that South Korea’s preemptive testing policy for long-term care facilities is effective [21].

Health system resilience is very important in the response to a global crisis such as the COVID-19 pandemic. It is also essential to expand laboratory diagnostic capabilities in the early stage of an epidemic to cope with a surge in demand [22]. Especially during the pandemic, clinical laboratories had to expand their testing capacities for massive screening and rapid reporting of accurate results [23]. Therefore, to reduce the resources required to handle a large number of specimens, various types of pooled tests have been proposed, and a high-throughput automation system has been introduced [24]. In South Korea, a test method of pooling five specimens was used. Pooled tests are appropriate for performing large-scale tests in a situation with low prevalence. However, the daily positive rate of pooled tests in March 2022 reached a maximum of 26.1%, which was relatively inefficient. If the prevalence is 15% or more, pooled tests can remain effective if the pooling size is reduced to 3, but it is not easy to convert the protocol or change the systems [25].

In GC Labs, human resources were continuously added as requests for pooled tests increased. Since December 2020, when the number of pooled tests increased dramatically, the increase in human resources accelerated (Fig. 6). As COVID-19 progressed, the skills of medical technicians improved, but the number of tests per person increased by 1.2 times, which was not significant. Work fatigue was high, so testing personnel had to be rotated, and many new laboratory personnel were put in, so it was not easy to continuously increase overall proficiency. In addition, the pooled test is not very efficient with respect to human resources management, and it was more challenging to secure efficiency as the prevalence increased. These factors may explain why the increase in proficiency was not significant during the period when the test was performed.

In automated equipment for sample mixing, it is not easy to position the arm for decapping or pipetting because of the various sizes of sample containers used in South Korea, and the cotton swab inside the container disturbs the pipet position sensor or sticks to the pipet and may cause contamination [24]. In addition, in the case of a dispensing system equipped with a decapped sample container, there is a disadvantage in that contamination of the original sample may occur because the inlet of the container is open. Overall, it took more than twice as long as a skilled medical technician to mix, making it a challenging method to use. To use a liquid handler for the mixing process, the length or diameter of the sample container must be standardized, and the problem of opening and closing the cap and handling the swab must be solved.

GC Labs introduced KingFisher nucleic acid extraction equipment (Thermo Fisher Scientific, Rocklin, CA, USA) with a short extraction time of 37 min. In addition, liquid handler equipment for automatically dispensing reagents or nucleic acids was applied during the test process. Additionally, some samples were processed using a fully automated molecular analyzer such as the Cobas 8800 system (Roche, Basel, Switzerland). To respond appropriately to this pandemic, quick and accurate reporting of results was of utmost importance. By introducing an automation system, manual errors could be minimized, and test processing speed could be increased.

Nevertheless, these efforts, including the allocation of various resources and expansion of the logistic network, were only possible because of the characteristics of ICLs, which can be dedicated to testing. Clinical laboratories in hospitals have also expanded their testing personnel and testing space in response to the pandemic, but primarily for the purpose of monitoring nosocomial infections and surveillance of hospitalized patients and their caregivers. And, most of the specimen collection for large-scale SARS-CoV-2 rRT PCR tests in South Korea was done at screening stations and temporary screening stations of public health centers. Therefore, ICLs, equipped with logistical networks for transporting specimens from public health centers nationwide and computer systems for result reporting, had to perform an active role. Hospitals had to dedicate resources such as medical staff and space to focus on the care of patients with COVID-19 or other diseases rather than testing [12].

The role of these ICLs may also be limited in countries or regions with limited healthcare resources, or where public or private infrastructure for pandemic response is difficult to build. In these cases, selective testing is less burdensome on the national healthcare system than mass testing [26]. However, regions or countries with high testing among them demand may want to carefully consider a decentralized testing approach, such as a mobile laboratory system or point-of-care testing with appropriate facilities.

The current study is limited in scope to the analysis of tests performed solely by GC Labs, thereby precluding the ability to account for results from other ICLs. Furthermore, the scope of the study was confined solely to the testing component among the triad of COVID-19 control measures in South Korea, which encompasses testing, contact tracing, and treatment.

## Conclusions

During the COVID-19 pandemic, ICLs handled nearly 90% of all tests under South Korea’s testing policy. As one of the leading ICLs in South Korea, GC Labs has also been performing robust testing. This study analyzed SARS-CoV-2 rRT-PCR tests requested by GC Labs from multiple perspectives. Nationally, active implementation of pooled tests proved to be a helpful method for large-scale testing. The preemptive periodic testing of high-risk facilities using pooled tests revealed a lower prevalence than the general population. ICLs actively responded to mass testing needs by deploying human resources, introducing new equipment, and expanding laboratory spaces and logistics networks. This study provides insights for establishing policies to address future infectious diseases and strategies for expanding the testing capacities of institutions such as ICLs.

## Data Availability

The datasets used and/or analysed during the current study are available from the corresponding author on reasonable request.

## Abbreviations

SARS-CoV-2 rRT PCR: SARS-CoV-2 real-time reverse transcription polymerase chain reaction
RAT: Rapid antigen test
EUA: Emergency use authorization
ICLs: Independent clinical laboratories
KDCA: Korea Diseases Control and Prevention Agency
KSLM: Korean Society of Laboratory Medicine
